# High rates of impaired quality of life and social and economic problems at 6 months after COVID-19-related ARDS

**DOI:** 10.1186/s44158-022-00048-5

**Published:** 2022-05-16

**Authors:** Michele Umbrello, Sara Miori, Andrea Sanna, Sergio Lassola, Elena Baruzzo, Daniele Penzo, Giovanni Pedrotti, Annamaria Perino, Angelo Colombo, Rocco Pace, Sandra Magnoni

**Affiliations:** 1 Department of Anesthesia and Intensive Care II, San Carlo Borromeo Hospital, ASST Santi Paolo e Carlo, Milano, Italy; 2 Department of Anesthesia and Intensive Care, Santa Chiara Hospital, Trento, Italy; 3 Center for Neurocognitive Rehabilitation (CeRiN), CIMeC, Trento University, Trento, Italy; 4 Department of Sociology and Social Research, Trento University, Trento, Italy; 5 Department of Anesthesia and Intensive Care, S. Maria del Carmine Hospital, Rovereto, Italy

**Keywords:** Health-related quality of life, COVID-19, Critical illness, ARDS, C-ARDS, Socio-economic status

## Abstract

**Purpose:**

Assess long-term quality of life (HR-QoL) and socio-economic impact in COVID-19-related ARDS (C-ARDS) survivors.

**Methods:**

C-ARDS survivors were followed up at 6 months in this prospective, cohort study. HR-QoL was assessed using SF-36 and EQ-5D-5L, and the socio-economic burden of COVID-19 was evaluated with a dedicated questionnaire. Clinical data were prospectively recorded.

**Results:**

Seventy-nine survivors, age 63 [57-71], 84% male, were enrolled. The frequency of EQ-5D-5L reported problems was significantly higher among survivors compared to normal, in mobility, usual activities, and self-care; anxiety and depression and pain were not different. SF-36 scores were lower than the reference population, and physical and mental summary scores were below normal in 52% and 33% of the subjects, respectively. In the multivariable analysis, prolonged hospital length of stay (*OR* 1.45; *p* 0.02) and two or more comorbidities on admission (*OR* 7.42; *p* 0.002) were significant predictors of impaired “physical” and “mental” HR-QoL, respectively. A total of 38% subjects worsened social relations, 42% changed their employment status, and 23% required personal care support.

**Conclusions:**

C-ARDS survivors have long-term impairment in HR-QoL and socio-economic problems. Prolonged hospital stay and previous comorbidities are risk factors for developing health-related issues.

**Supplementary Information:**

The online version contains supplementary material available at. 10.1186/s44158-022-00048-5

## Introduction

Ever since its first description in late 2019, the global pandemic caused by the new SARS-COV-2 virus and the associated COVID-19 disease has caused > 360 million confirmed cases and > 5.6 million deaths. The clinical spectrum of COVID-19 ranges from asymptomatic cases to highly aggressive respiratory failure and ARDS (C-ARDS), which often leads to rapid and unexpected deterioration and significant mortality even in previously healthy and fully active people [1, 2].

It is increasingly acknowledged how patients who survive critical illness commonly report persistent physical and cognitive impairments on their health-related quality of life (HR-QoL), a reduced ability to care for themselves, to perform usual activities of daily living, and to participate in social roles as compared to an age- and gender-matched population [3–5]. Despite a huge amount of research on the pathophysiology and management of critically ill patients with COVID-19, only a few studies have assessed post-intensive care discharge persistent symptoms and HR-QoL [6–10].

All-cause ARDS survivors demonstrated a marked reduction in nearly all physical and mental domains of HR-QoL assessments in the post-discharge period compared with a reference population [11, 12]. Previous data after the Middle East respiratory syndrome (MERS) outbreak found that neither physical nor mental HR-QoL scores were different between patients with MERS and non-MERS acute respiratory failure; however, critically ill MERS survivors reported lower HR-QoL than survivors who had not been admitted to the ICU [13]. A recent telephone, follow-up study on 120 COVID-19 patients showed that most patients had persistent symptoms 4 months after discharge, especially fatigue and dyspnea [8]. In a large cohort study with 6 months follow-up, up to 75% of COVID-19 survivors reported at least one symptom, mainly fatigue or muscle weakness, sleep difficulties, and anxiety or depression, as well as a reduction in the overall HR-QoL [9].

Besides the effects of critical illness on the perceived physical and mental health, also the socio-economic determinants and consequences of critical illness need to be considered. A recent exploratory study identified a significant socio-economic burden following critical illness; a cohort of critical illness survivors showed functional disabilities, faced a negative impact on employment, and commonly had a care requirement after discharge from hospital, with a corresponding reduction in their level of income [14]. Indeed, limited attention has been paid on the extent to which the socio-economic status is linked to the health conditions and the outcomes of COVID-19. According to the extensive literature on the social shaping of health and disease, patients with reduced economic, social, and cultural resources are more likely to experience worse health conditions and lower quality of life, as health is exclusively not the product of individual characteristics but also the outcome of the contextual dimension in which people are embedded [15, 16], and patients with a higher socio-economic status show a greater functional recovery after critical illness [17]. During the COVID-19 outbreak, morbidity and mortality were found to be related to the socio-economic characteristics of local areas both in the UK [18] and in the 50 largest US cities [19]. Similarly, socio-economic indicators such as the educational disadvantage, unemployment, and population density were significantly associated with the incidence and outcome of COVID-19 in northern Italy, suggesting a pattern of socio-economic inequalities in the outbreak [20].

The aim of this study is to (1) assess the quality of life in a cohort of patients with C-ARDS 6 months after intensive care discharge and identify long-lasting physical and psychological symptoms impairing normal daily activities, (2) identify social and disease-related risk factors for long-term health consequences, and (3) recognize changes in relational, work, and economic skills, quantify any unrecognized need for health, and assess the responses of the national health service.

## Materials and methods

### Participants and study design

**Fig. 1:**
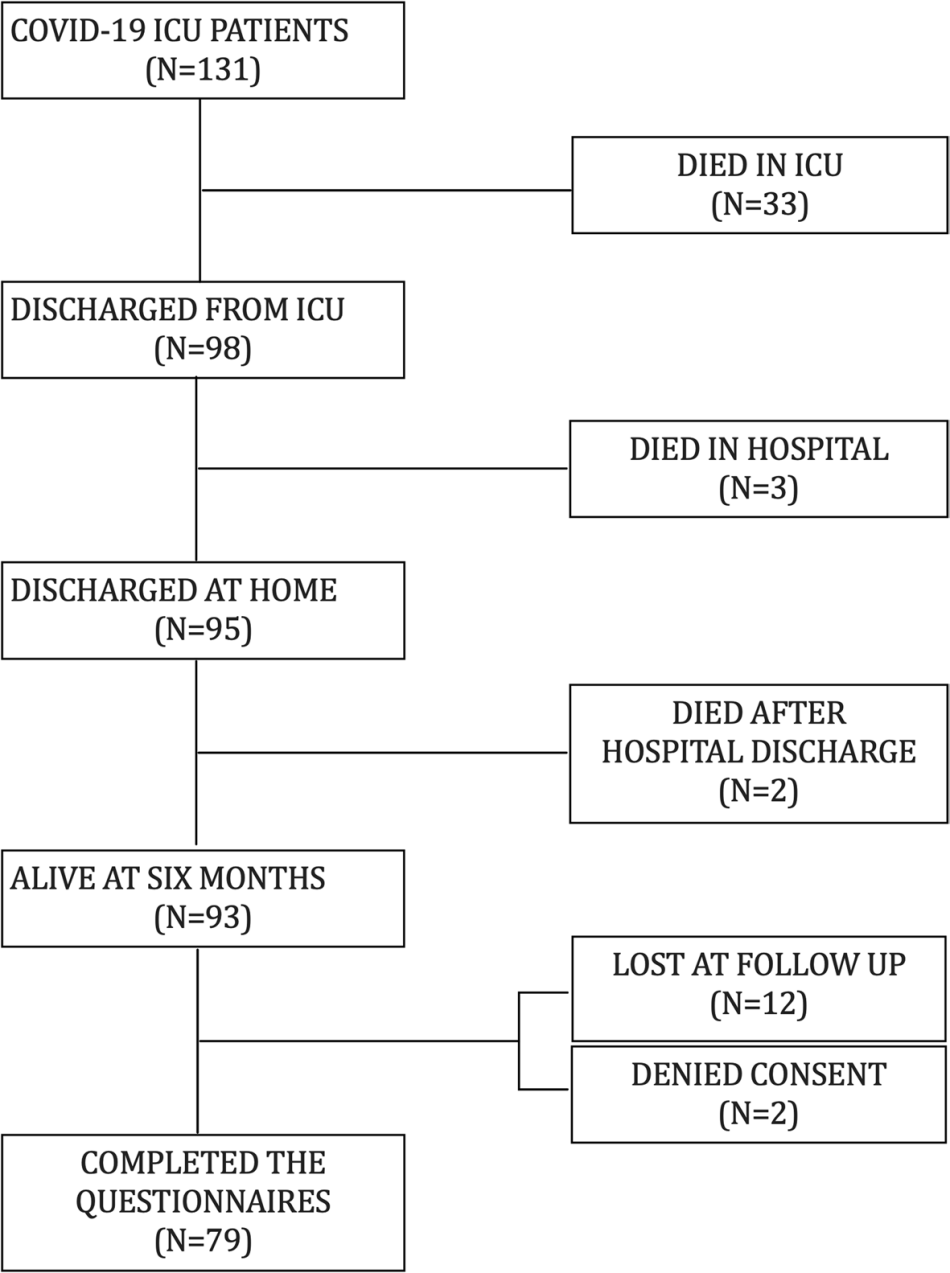
Study flow chart

Consecutive patients admitted to the ICUs of two distinct referral hospitals in the northeast Italian region Trentino-Alto Adige (S. Chiara Hospital-Trento and S. Maria del Carmine Hospital-Rovereto) between March 5 and April 30, 2020, were screened for enrollment in this prospective, cohort study investigating the impact of COVID-19 on the quality of life and the socio-economic status. Adult (> 18 years), Italian-speaking patients admitted to ICU (> 24 h) for COVID-19 ARDS, according to the Berlin definition [21], were included. Those who did not respond at follow-up or denied participating were excluded. A flow chart of the study is shown in Fig. 1.

The local ethics committee approved the study, as part of a follow-up study on COVID-19 survivors (APSS; protocol number 1/2021), including a waiver of consent for hospital data collected during the pandemic surge (APSS; protocol number 3/2020). Six months after ICU discharge, COVID-19 ICU survivors were firstly contacted by experienced ICU physicians (AS, SM) with an informative phone call, followed by an email with a brief description of the study. Patients were asked by email for their consent to participate to the study and appointed for a telephone interview. Since restrictions due to the pandemic prevented the possibility to perform face-to-face interviews, the follow-up was performed on the telephone, by an experienced ICU physician previously involved in patient clinical management.

At the time of the structured telephone interview, patients were administered a set of questionnaires including the five-dimension, five-level EuroQoL questionnaire (EQ-5D-5L), the EuroQol Visual Analogue Scale (EQ VAS), and the Short Form Health Survey 36, version 2 (SF-36v2) to assess the HR-QoL, a novel question set designed to determine changes in family circumstances, socio-economic status, and care requirements. The duration of the interview was approximately 45 min.

### EQ-5D-5L and EQ VAS

The EQ-5D-5L scale describes 5 dimensions: mobility, self-care, usual activities, pain/discomfort, and anxiety/depression [22]. Each dimension has 5 levels: 1 no problems, 2 slight problems, 3 moderate problems, 4 severe problems, and 5 extreme problems. The respondent is asked to indicate his/her health state in each of the 5 dimensions. Based on the five answers, a summary EQ-5D-5L index was calculated using a recently published value set for the Italian people, with values ranging from −0.571 for the worst health state (55,555) to 1 for the best health state (11,111). As a reference value, we used mean 0.91 (SD 0.14) for an age-matched population [23].

The EQ VAS records the respondent’s self-rated health on a vertical, visual analogue scale where the endpoints are labeled “Best imaginable health state” and “Worst imaginable health state.” This information can be used as a quantitative measure of health outcome as judged by the individual respondents.

### SF-36 version 2

The SF-36 is an 11-question, 36-item questionnaire frequently used to describe HR-QoL after ICU [24, 25]. It investigates 8 health domains: physical functioning, the extent to which health limits physical activity; physical role, the extent to which physical health interferes with work or limits activity; pain, the intensity of pain and the effect of pain on patient’s ability to work; general health, patient’s perception of his or her health; vitality, the degree of energy the patient has; social functioning, the extent to which health or emotional problems interfere with social activities; emotional role, the extent to which emotional problems interfere with work or activities; and mental health, general mental health. Estimate scores for the 8 domains were calculated as recommended in the specific manual and interpretation guide. The overall score on each SF-36 subscale ranges from 0 to 100 with higher scores indicating a better HR-QoL. We also calculated the SF-36 physical (PCS) and mental (MCS) component summary measures by combining the subscale scores physical and mental components, as recommended in the specific manual [26]. A reference level of 50 (SD 10) points was adopted for both components, as calculated for the US population, and validated in Australia, France, and Italy [26]. Patients were then classified into two groups based on physical and mental components as follows: (1) a reduced HR-QoL if the score was lower than 1 SD below the mean reference value (i.e., < 40) and (2) normal HR-QoL if the score was higher than 1 SD below the mean reference value (i.e., > 40).

### Socio-economic questionnaire

The socio-economic questionnaire consists of a multiple-choice questionnaire to evaluate the socio-economic-relational impact on patients and families, previously used in general intensive care survivors in the UK and subsequently adapted to the Italian context with the help of a health sociologist (prof. Annamaria Perino, Department of Sociology and Social Research, University of Trento, Italy), who validated the socio-economic question set [14]. In detail, we collected the following: demographic and socio-economic data about patients and family, economic and socio-relational impact of the disease and the ICU stay, information on health service utilization and need for care after ICU discharge, and observed possible stigmatization processes of the survivor and of their families.

### Clinical measures

Epidemiological and clinical information about ICU stay, severity scores, and laboratory data was manually or digitally extracted and recorded from our electronic system. All data were anonymized and saved in Microsoft Excel files. Education levels were classified according to the International Standard Classification of Education (ISCED): ISCED 0, early childhood education (“less than primary” for educational attainment); ISCED 1, primary education; ISCED 2, lower secondary education; ISCED 3, upper secondary education; ISCED 4, postsecondary non-tertiary education; ISCED 5, short-cycle tertiary education; ISCED 6, bachelor’s or equivalent level; ISCED 7, master’s or equivalent level; and ISCED 8, doctoral or equivalent level.

### Statistical analysis

Descriptive results for continuous variables are reported as mean (SD) if normally distributed, or median (25th–75th percentiles) otherwise, and as count and percentages when indicated. Variable distribution was tested by the Kolmogorov–Smirnov and Shapiro–Wilk tests.

As social and disease-related risk factors, eight clinically and epidemiologically relevant variables (i.e., age, education, income, comorbidities, worst PaO_2_/FiO_2_ ratio on the first ICU day), SAPSII (simplified Acute Physiology Score), days of artificial ventilation with PEEP (either invasive or noninvasive), and length of hospital stay (LOS) were selected a priori and dichotomized using meaningful reference values, as follows: age ≤ or >65 years (as relevant to COVID-19 [27]), education ISCED ≤ or > 2 (identifying low or medium/high education), income ≤ or >1500 € (as the median value), comorbidities ≤ or > 1 (as relevant to COVID-19 [27]), PaO_2_/FiO_2_ ratio ≤ or > 150 mmHg (as performed in previous ARDS studies [28]), SAPS II ≤ or > 25 (as the median value), and artificial ventilation and hospital stay, respectively, ≤ or > 15 days and 4 weeks (as relevant to COVID-19 [29]). Each of the EQ-5D-5L dimensions were grouped in two categories: “no problems” and “some problems,” this latter including slight, moderate, severe, and extreme problems, if applicable. Mann Whitney *U*-test was used for comparisons of non-normally distributed continuous variables, one-sample *t*-test was used to compare SF-36 dimensions and EQ-5D-5L summary index of survivors with normative data from the general population, and Fisher test was used to analyze categorical variables between groups.

Univariate binomial logistic regression analysis was used to explore risk factors (covariates) associated with each of the EQ-5D-5L domains (dependent variable): pain, mobility, usual activity, self-care problems, and anxiety and depression. Reference value for the dependent variables was set as follows: no problems vs. some problems. Continuous variables were transformed as follows: age/10, PaO_2_/FiO_2_/100, SAPSII/5, duration of ventilation (days)/10, and hospital LOS (days)/10. Categorical variables (education, income, and comorbidities) were dichotomized as previously described. Wald test was used for regression analysis.

Multivariable binomial logistic regression analysis was used to explore associated factors with reduced HR-QoL, assessed by SF-36 PCS and MCS < 40. For model selection, we used a stepwise selection approach based on the Akaike information criterion (AIC) and calculated the adjusted odds ratios with 95% confidence intervals.

To account for the presence of both patients who only received noninvasive mechanical ventilation, as opposed to patients who were invasively ventilated, two sensitivity analyses were performed; we compared the demographic, socio-economic, and clinical characteristics of the two subgroups of patients, and we repeated all the analyses only in the subgroup of invasively ventilated patients.

Statistical analysis was performed with jamovi (the jamovi project 2012; version 1.6) and R version 4.0.2. GraphPad software Prism (version 8.4.3, 2020) was used for graphical representation. For all the comparison, *p* < 0.05 was considered statistically significant.

## Results

### Characteristics of the patients

Of the 93 potentially recruitable patients, 79 were included in the study (Fig. 1). Clinical and demographic data are described in Table 1. The median age of the patients was 63 years, and 84% were male, 54% were pensioners, and 65% had an ISCED level > 2; most of the patients lived in small towns (< 15,000 inhabitants) as a reflection of the geographical composition of a rural territory. A total of > 75% of the patients had no or only one preexisting comorbidity, mostly hypertension (46%). All patients had ARDS, 69 (87%) required invasive mechanical ventilation, and 10 (13%) received noninvasive ventilation with PEEP ≥ 5 cmH_2_0. Laboratory data and information about ICU treatments are summarized in Table 1. The characteristics of the noninvasively ventilated C-ARDS patients and the comparison with the mechanically ventilated patients are shown in Supplementary Table S1. Despite similar gas exchange derangement on ICU admission, noninvasively ventilated patients had a higher BMI, received no or less rescue therapies, and had a shorter ICU and hospital stay.

**Table 1:** Demographic, socio-economic, and clinical characteristics of C-ARDS survivors responding at follow-up

**Demographic characteristics**	
Number of patients	79
Age (years)	63 (57–71)
Sex	
Male	66 (84%)
Female	13 (16%)
**Socio-economic characteristics**	
Family unit composition	
Single	9 (11%)
≥ two people	70 (89%)
Population of the municipality of residence	
< 15,000	66 (84%)
> 100,000	13 (16%)
Employment status^a^	
Active worker	33 (42%)
Retired	43 (54%)
Monthly income (€)	
≤ 1500	41 (54%)
> 1500	35 (46%)
Education	
ISCED 0–2	27 (34%)
ISCED > 2	51 (65%)
**Clinical characteristics**	
No preexisting comorbidities	38 (48%)
Preexisting comorbidities	41 (52%)
One comorbidity	23 (29%)
More than one comorbidity	18 (23%)
Comorbidities	
Hypertension	36 (46%)
Diabetes	9 (11%)
Asthma and/or COPD	10 (13%)
Ischemic heart disease	6 (8%)
Neoplasm	4 (5%)
Chronic liver or kidney disease	
Immunocompromised	
BMI (kg/m^2^)	27 (25–29)
Time from symptoms to hospital admission (days)	7 (5–10)
SOFA score on ICU admission	6 (4–7)
SAPS II score on ICU admission	27 (24–33)
PaO_2_/FiO_2_ on ICU admission (worst)	160 (114–225)
Renal replacement therapy	5 (6%)
Prone positioning	35 (44%)
Hydroxychloroquine	76 (97 %)
Steroids	37 (47%)
Tocilizumab	16 (20%)
Tracheostomy	22 (28%)
Duration of ventilation (days)	16 (12–25)
ICU LOS (days)	20 (13–28)
Hospital LOS (days)	40 (29–49)
**Laboratory data in the ICU**	
Creatinine on admission (mg/dl)	0.92 ± 0.38
Creatinine max (mg/dl)	2.00 ± 2.15
D-dimer on admission (μg/mL)	2407 ± 5892
D-dimer max (μg/mL)	5821 ± 9694
WBC on admission (10^3/mL)	9.47 ± 4.42
WBC max (10^3/mL)	17.28 ±7.6
CRP on admission (mg/dl)	113 ± 81
CRP max (mg/dl)	234 ± 114

Values are presented as median (25–75th percentile), mean ± SD, or as count (percentage) for categorical variables

*BM*I body mass index, *SOFA* Sequential Organ Failure Assessment Score, *SAPS II* Simplified Acute Physiology Score, *RRT* renal replacement therapy; duration of ventilation (days), days of ventilation with PEEP ≥ 5 cmH_2_0, *ICU LOS* length of stay in intensive care unit, *ISCED* International Standard Classification of Education, *ISCED 0* early childhood education (“less than primary” for educational attainment), *ISCED 1* primary education, *ISCED 2* lower secondary education, *ISCED 3* upper secondary education, *ISCED 4* postsecondary non-tertiary education, *ISCED 5* short-cycle tertiary education, *ISCED 6* bachelor’s or equivalent level, *ISCED 7* master’s or equivalent level, *ISCED 8* doctoral or equivalent level, *WBC* white blood cells, *CRP* C-reactive protein

^a^Housewives excluded (*N* = 3)

### Quality-of-life assessment

#### EQ-5D-5L

**Fig. 2:**
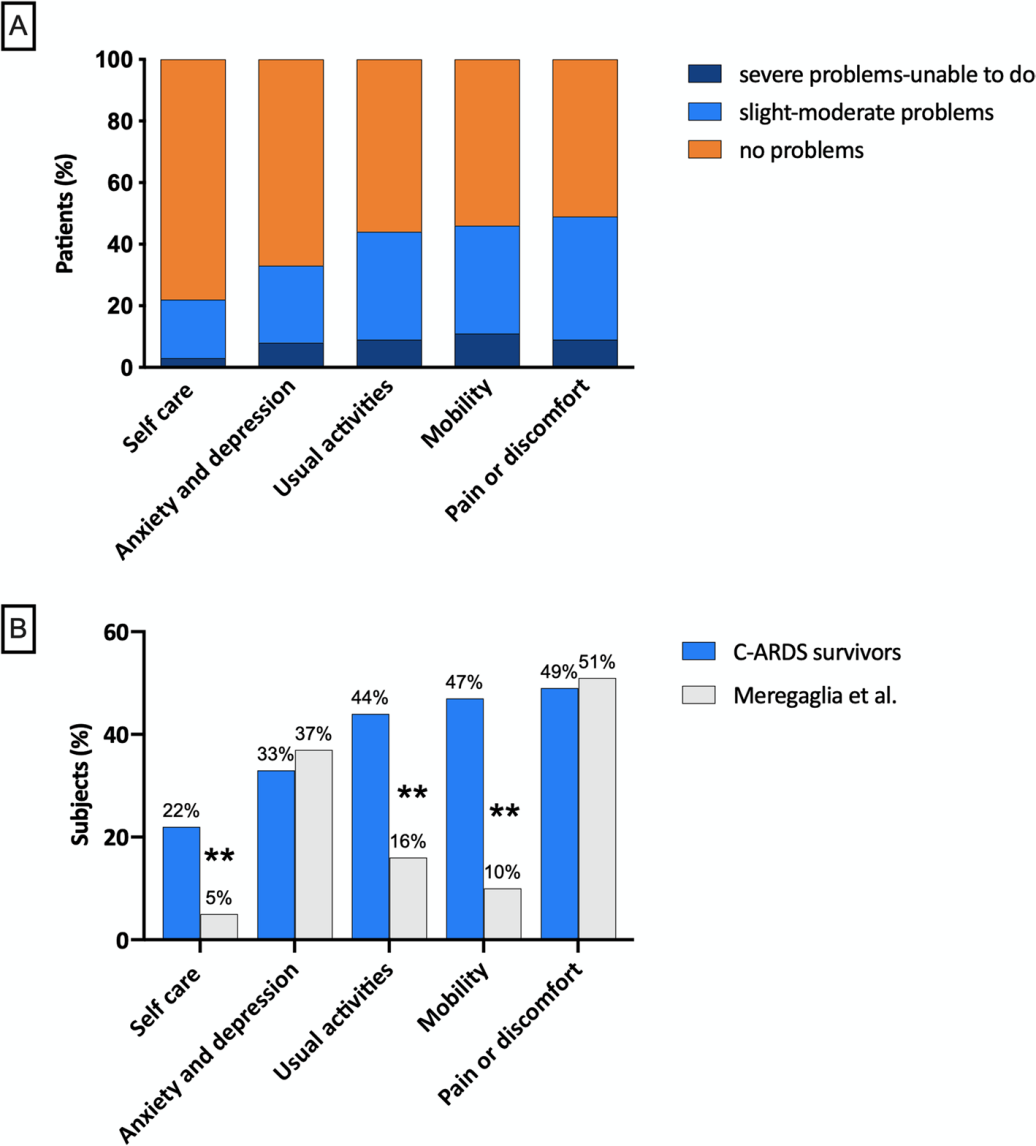
Health-related quality-of-life scale (EQ-5D-5L) in C-ARDS survivors. **A** Frequency distribution of the EQ-5D-5L scores in each of the five domains (pain or discomfort, mobility, usual activities, anxiety, and depression, self-care). Each domain is scored on a 5-point scale: 1 no problems, 2 slight problems, 3 moderate problems, 4 severe problems, and 5 unable to do. Scores are grouped in three classes: no problems, slight to moderate problems, and severe problems or unable to do. **B** EQ-5D-5L domain scores for patients responding at the structured interview compared to Italian norm data. Data represent the percentage of subjects and patients with problems of any level (2 to 5 in each of the five domains). Significant differences between C-ARDS survivors (*N* = 79) and age-matched Italian subjects [30] (*N* = 211) were analyzed using Fisher’s exact test. **p < 0.0001

HR-QoL, as assessed by the EQ-5D-5L, and compared to Italian reference levels, is summarized in Fig. 2. The majority of subjects reported slight or moderate problems, whereas < 10% reported a severe health impairment (i.e., not able to do things). Full health (no problems in any domain) was disclosed in 19 (24%) patients, whereas problems in one or two domains were detected in 32 (41%), and worse health conditions with problems in three, four, or all five domains were identified in 28 (35%) patients. The proportion of patients reporting problems of any severity (level 2 to 5) was significantly higher in C-ARDS survivors compared to age-matched Italian norm data, in mobility, usual activities, and self-care; anxiety and depression and pain were not different [30]. The mean EQ-5D-5L summary index of C-ARDS survivors was 0.798 (SD 0.288) vs. 0.91 (SD 0.14) in the age-matched Italian population (one-sample t-test; *p* = 0.001). The average EQ VAS score was 80 [60-89] vs. a reference age-matched population of 80 [70-90]. Equivalent results were obtained in the subgroup of invasively ventilated C-ARDS patients (Supplementary Fig. S1).

#### SF-36

SF-36 analysis shows lower values in most of the domain scores, except for pain, emotional role, and mental health, when compared with Italian norms (Table 2). The average SF-36 physical (PCS) and mental component summary scores (MCS) were 43.6 (34.4–50.2) and 52.1 (44.5–57.0). Single patients’ summary scores were below normal in 52% of the subjects for the PCS and in 33% of the patients for the MCS.

**Table 2:** SF-36 domain scores of C-ARDS survivors compared to Italian normalized data

SF-36 domains	COVID-19 survivors (*N* = 79)	Apolone et al. (*N* = 2031)	*p*
Physical function	**78.22** ± 25.59	**84.46** ± 23.18	0.0197
Physical role	**48.10** ± 48.00	**78.21** ± 35.93	< 0.0001
Bodily pain	**73.92** ± 28.77	**73.67** ± 27.65	0.9373
General health	**43.79** ± 26.03	**65.22** ± 22.18	< 0.0001
Vitality	**57.03** ± 20.64	**61.89** ± 20.69	0.0405
Social function	**64.40** ± 33.44	**77.43** ± 23.34	< 0.0001
Emotional role	**76.79** ± 40.07	**76.16** ± 37.25	0.8831
Mental health	**72.91** ± 19.31	**66.59** ± 20.89	0.0082

The normal Italian values are from Apolone et al. [25]. Data are represented as mean ± SD. Differences between SF-36 scores in the COVID-19 survivors and the Italian reference population were analyzed using one-sample t-test

### Factors associated with impaired quality of life

#### EQ-5D-5L

**Fig. 3:**
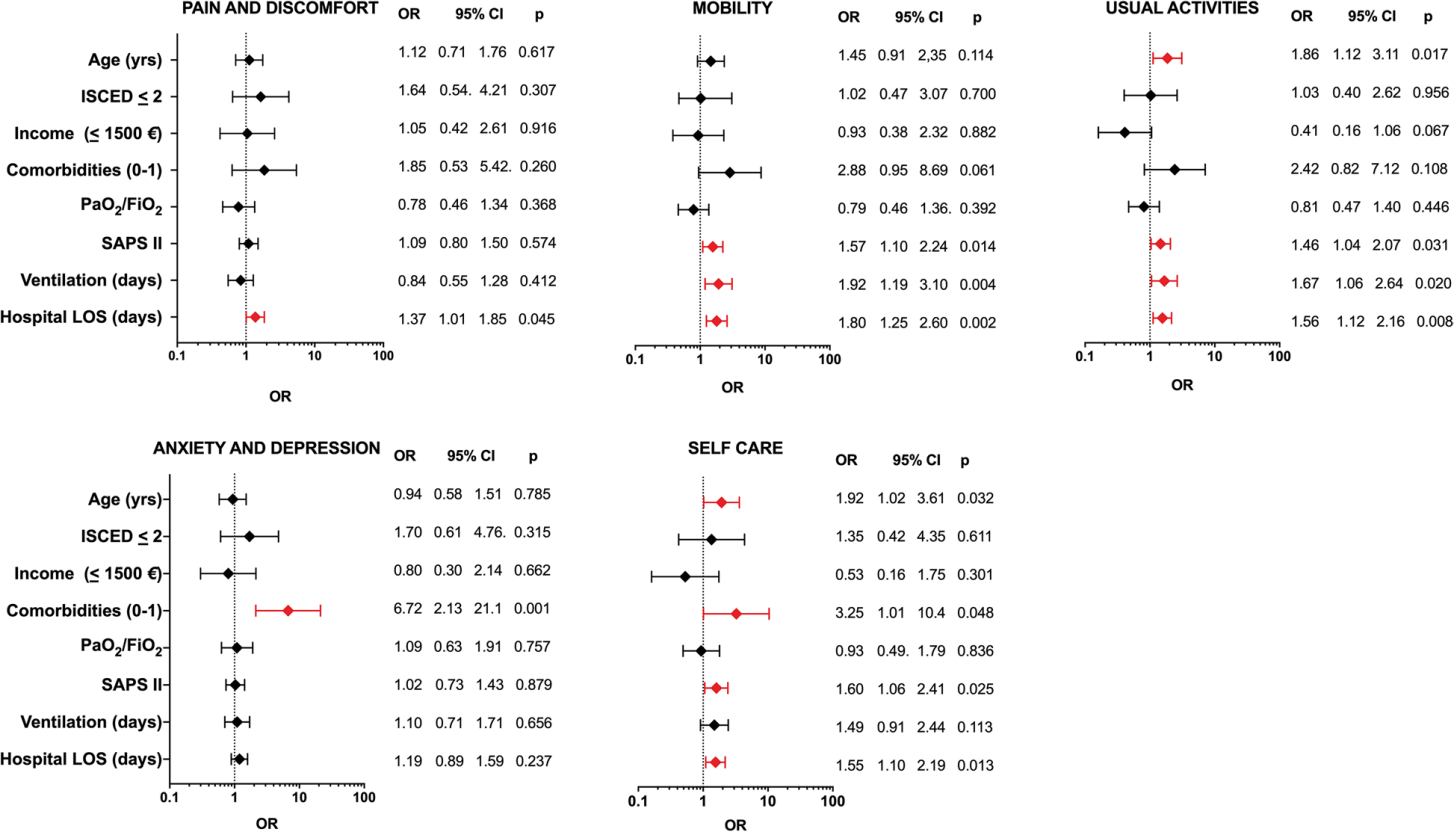
Univariate analysis of demographics, socio-economic, and clinical factors associated with impaired health-related quality of life (EQ-5D-5L) in C-ARDS survivors. The figure shows the results of the univariate analysis of factors associated with problems (of any severity) in each of the five EQ-5D-5L domains (dependent variable) and the correspondent forest plots. In red, variables significantly associated with the development of problems in each single domain. Reference value for the dependent variable: no problems vs any problems. Covariate values are on the y-axis, with reference values and/or unit of measure, when applicable. Variables were transformed as follows: age/10, PaO_2_ to FiO_2_/100, SAPS II/5, duration of mechanical ventilation (days)/10, and hospital LOS (days)/10. SAPS II, Simplified Acute Physiology Score; ventilation (days), duration of ventilation with PEEP ≥ 5 cmH_2_0; LOS, length of stay

Epidemiological and clinical variables associated at the univariate analysis with impairment (any problems vs. no problems), in each of the five EQ-5D-5L domains in C-ARDS survivors, are summarized in Fig. 3. Length of hospital stay was associated with impairment in most of the domains (pain and discomfort, mobility, usual activities, and self-care). Increased SAPS II was also associated with problems in several domains (mobility, usual activities, and self-care), and two or more comorbidities on admission, increased age, and duration of ventilation were associated with problems in two domains. The results of the analysis in the subgroup of invasively ventilated C-ARDS patients were equivalent, with hospital length of stay and > 1 comorbidities being the most frequent factors associated with impaired quality of life (Supplementary Table S2).

#### SF-36

Table 3 shows SF-36 domain scores in C-ARDS survivors according to the eight epidemiological and clinical variables. Patients with a high burden of comorbidities (compared to those with no or only one comorbidity) had reduced scores in almost all SF-36 domains (seven out of eight); lower scores were also found in patients with worse gas exchanges (PaO_2_/FiO_2_ ≤ 150) on ICU admission, longer duration of ventilation, or prolonged hospital stay (four out of eight domains) and in patients with SAPS II score > 25 (two out of eight domains). No significant differences were found in regard to age, sex, education, family income, and utilization of rescue therapies such as prone positioning, laboratory data (i.e., CRP), or renal replacement therapies (data not shown). Equivalent results were found in the subgroup of invasively ventilated C-ARDS patients (Supplementary Table S3).

**Table 3:** SF-36 domain scores according to demographics, socio-economic, and clinical characteristics in C-ARDS survivors

			***Physical function***	***Physical role***	***Bodily pain***	***General health***	***Vitality***	***Social function***	***Emotional role***	***Mental health***
**Age (years)**	< 65	43	**90**	**75**	**90**	**40**	**65**	**75**	**100**	**80**
			(80–98)	(0–100)	(68–100)	(25–60)	(53–70)	(50–88)	(67–100)	(70–88)
	> 65	36	**85**	**13**	**76**	**48**	**55**	**63**	**100**	**78**
			(64–95)	(0–100)	(45–100)	(20–70)	(40–71)	(25–100)	(67–100)	(56–88)
	*p*		*p* = 0.2158	*p* = 0.5171	*p* = 0.1615	*p* = 0.6*715*	*p* = 0.3882	*p* = 0.4159	*p* = 0.9152	*p* = 0.5626
**Education (ISCED)**	0–2	28	**85**	**50**	**90**	**50**	**60**	**88**	**100**	**84**
			(80–98)	(0–100)	(68–100)	(20–70)	(45–78)	(50–100)	(100–100)	(64–92)
	> 2	51	**85**	**25**	**78**	**45**	**55**	**63**	**100**	**76**
			(63–95)	(0–100)	(45–100)	(25–60)	(45–70)	(25–88)	(33–100)	(64–88)
	*p*		*p* = 0.3815	*p* = 0.9121	*p* = 0.0920	0.4841	*p* = 0.5873	*p* = 0.1362	*p* = 0.1990	*p* = 0.2109
**Monthly income (€)**	< 1500	41	**85**	**0**	**88**	**45**	**55**	**63**	**100**	**80**
			(80–95)	(0–100)	(50–100)	(25–60)	(45–70)	(25–88)	(67–100)	(68–88)
	> 1500	35	**90**	**100**	**78**	**50**	**65**	**75**	**100**	**80**
			(70–98)	(0–100)	(55–100)	(20–75)	(50–70)	(56–100)	(83–100)	(64–88)
	*p*		*p* = 0.7281	*p* = 0.1537	*p* = 0.8466	*p* = 0.2239	*p* = 0.3575	*p* = 0.1844	*p* = 0.7816	*p* = 1.000
**Comorbidities**	0–1	61	**90**	**50**	**90**	**50**	**65**	**88**	**100**	**80**
			(80–100)	(0–100)	(68–100)	(30–70)	(50–75)	(50–100)	(100–100)	(68–88)
	> 1	18	**70**	**0**	**50**	**15**	**45**	**31**	**0**	**62**
			(4–90)	(0–100)	(25–85)	(5–45)	(26–55)	(13–75)	(0–100)	(45–80)
	*p*		*p* = 0.0037^a^	*p* = 0.2446	*p* = 0.0018^a^	*p* = 0.004^a^	*p* = 0.0010^a^	*p* = 0.0021^a^	*p* < 0.0001^a^	*p* = 0.0017^a^
**PaO**_**2**_**/FiO**_**2**_ **on ICU admission**	< 150	34	**83**	**0**	**78**	**33**	**55**	**56**	**100**	**80**
			(65–95)	(0–100)	(38–100)	(16–58)	(41–69)	(25–88)	(0–100)	(58–88)
	> 150	44	**90**	**100**	**90**	**48**	**60**	**88**	**100**	**76**
			(80–96)	(0–100)	(55–100)	(29–71)	(50–71)	(50–100)	(0–100)	(64–88)
	*p*		*p* = 0.1007	*p* = 0.0388^a^	*p* = 0.0529	*p* = 0.0313^a^	*p* = 0.1276	*p* = 0.0225^a^	*p* = 0.0330^a^	*p* = 0.4059
**SAPS II**	< 25	28	**90**	**75**	**90**	**38**	**63**	**75**	**100**	**78**
			(85–100)	(0–100)	(68–100)	(16–54)	(55–74)	(50–88	(67–100)	(62–88)
	> 25	51	**85**	**25**	**76**	**50**	**55**	**75**	**100**	**80**
			(65–95)	(0–100)	(45–100)	(25–70)	(45–70)	(25–100	(67–100)	(64–88)
	*p*		*p* = 0.0202^a^	*p* = 0.2835	*p* = 0.0363^a^	0.9283	*p* = 0.1268	*p* = 0.1481	*p* = 0.4662	*p* = 0.5614
**Ventilation (days)**	≤ ***15***	36	**90**	**100**	**90**	**45**	**63**	**88**	**100**	**82**
			(80–100)	(0–100)	(64–100)	(25–71)	(54–80)	(50–100)	(100–100)	(71–88)
	> 15	43	**85**	**0**	**78**	**45**	**55**	**63**	**100**	**72**
			(63–95)	(0–100)	(48–100)	(20–61)	(38–70)	(31–88)	(33–100)	(58–88)
	*p*		*p* = 0.0388^a^	*p* = 0.0043^a^	*p* = 0.0396^a^	*p* = 0.2556	*p* = 0.0500	*p* = 0.0187^a^	*p* = 0.0672	*p* = 0.0650
**Hospital LOS (weeks)**	≤ 4	23	**90**	**100**	**100**	**45**	**60**	**88**	**100**	**84**
			(85–100)	(0–100)	(89–100)	(20–63)	(55–80)	(50–100)	(100–100)	(70–88)
	> 4	56	**85**	**0**	**73**	**45**	**58**	**75**	**100**	**76**
			(64–95)	(0–100)	(45–100)	(20–65)	(40–70)	(25–88)	(33–100)	(59–88)
	*p*		*p* = 0.0344^a^	*p* = 0.0112^a^	*p* = 0.0025^a^	*p* = 0.4634	*p* = 0.0411^a^	*p* = 0.0945	*p* = 0.0806	*p* = 0.1061

SF-36 scores for each domain can range from 0 to 100; higher scores correspond to a better health-related quality of life. Data are espressed as median (25th–75th) percentile of SF-36 domains score and analyzed with Mann-Whitney *U*-test; ^a^corresponds to significant differences between groups; *p* < 0.05 was considered significant. *ISCED*, International Standard Classification of Education. *ISCED 0*, early childhood education (“less than primary” for educational attainment)

*ISCED 1* primary education, *ISCED 2* lower secondary education, *ISCED 3* upper secondary education, *ISCED 4* postsecondary non-tertiary education, *ISCED 5* short-cycle tertiary education, *ISCED 6* bachelor’s or equivalent level, *ISCED 7* master’s or equivalent level, *ISCED 8* doctoral or equivalent level, *ICU* intensive care unit, *SAPS II* Simplified Acute Physiology Score, *LOS* length of stay; ventilation (days), duration of ventilation with PEEP ≥ 5 cmH_2_0

**Fig. 4:**
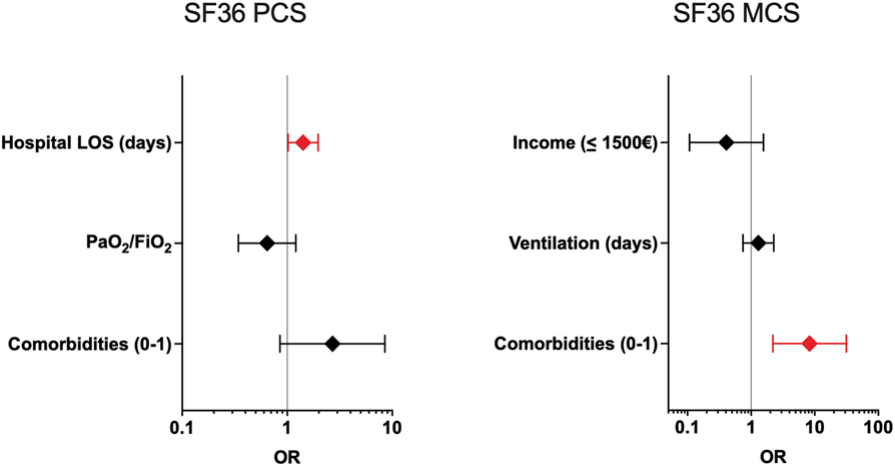
Multivariable analysis of factors associated with impaired “physical” health-related quality-of-life scale (SF-36 PCS) and “mental” health-related quality-of-life scale (SF-36 MCS) in C-ARDS survivors. The figure shows factors associated with SF-36 physical component summary score (PCS) and SF-36 mental component summary score (MCS) < 40 (dependent variable), the calculated adjusted odds ratios, and the 95% confidence intervals, based on the logistic regression, on the x-axis, and on the correspondent forest plot. In red, variables significantly associated with an impaired quality of life. Reference values for the dependent variable: PCS and MCS ≥ 40. Covariate values are on the y-axis, with reference values, when applicable (factors). Variables were transformed as follows: PaO_2_ to FiO_2_/100, ventilation (days)/10, hospital LOS (days)/10; ventilation (days): duration of ventilation with PEEP > 5 cmH_2_0; *LOS*: length of stay

A multivariable analysis identified the predictors that were best associated with an impaired quality of life in C-ARDS survivors, as reflected by physical and mental component summary scores (PCS and MCS) below 40. Predictors associated with reduced PCS were hospital length of stay [*OR* 1.42 (95% *CI* 1.02–1.97); *p* = 0.02], two or more comorbidities on admission [*OR* 2.69 (95% *CI* 0.85–8.53); *p* = 0.088], and worse PaO_2_/FiO_2_ on ICU admission [*OR* 0.64 (95% *CI* 0.34–1.21); *p* = 0.16] (Fig. 4); two or more comorbidities on admission were the only significant predictors associated with reduced MCS [*OR* 8.30 (95% *CI* 2.19–31.46); *p* = 0.001] in the model, which also included income [*OR* 0.40 (95% *CI* 0.11–1.56); *p* = 0.18] and the duration of ventilation [*OR* 1.29 (95% *CI* 0.74–2.27); *p* = 0.36]. Equivalent results were found in the cohort of invasively ventilated C-ARDS survivors, where hospital length of stay [*OR* 1.49 (95% *CI* 1.02–2.18); *p* = 0.02] and two or more comorbidities on admission [*OR* 5.75 (95% *CI* 1.41–23.33); *p* = 0.014] were the only significant predictors of impaired PCS and MCS, respectively (Supplementary Fig. S2).

### Social relations and requirement of care

**Fig. 5:**
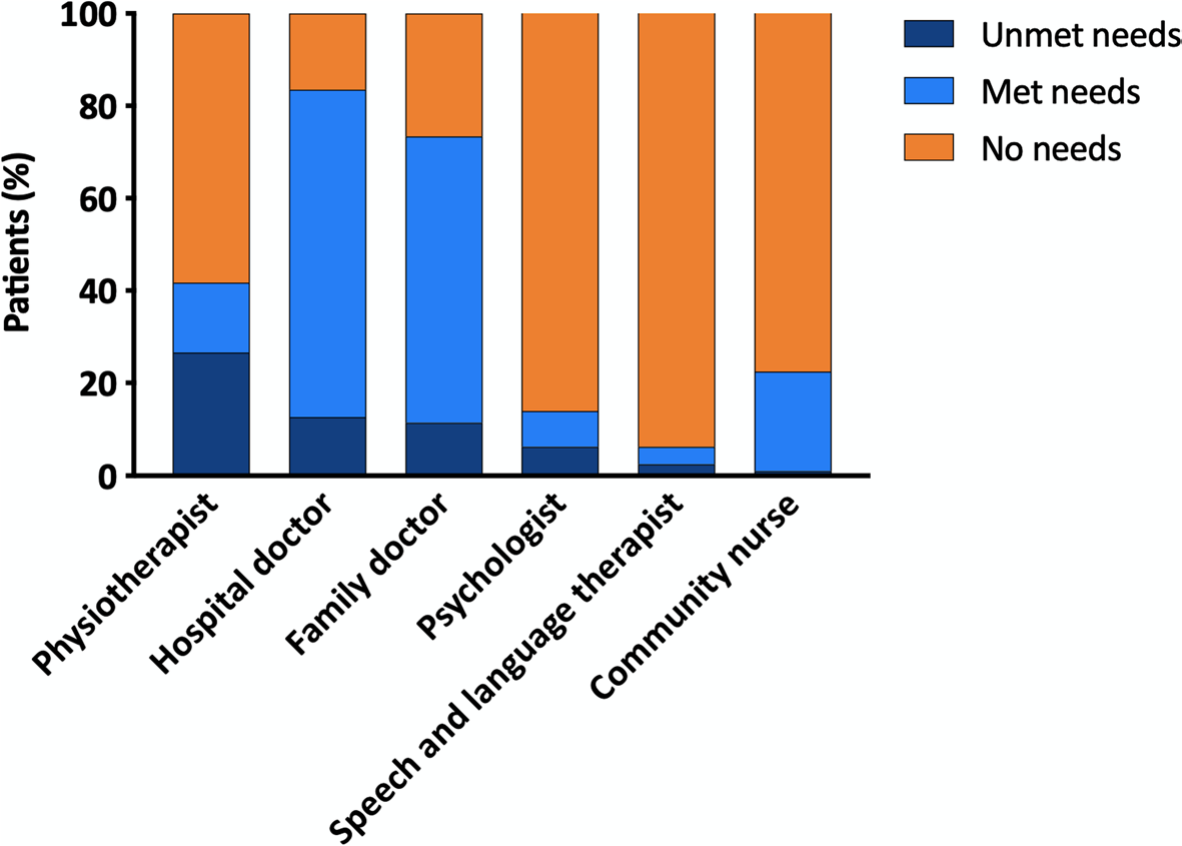
Description and distribution of health professionals needs in C-ARDS survivors

Social and relational life were affected by C-ARDS in a significant proportion of subjects. Table 4 shows that 38% of the patients were negatively affected in their relationships, 58% of the active workers had returned to full-time work after 6 months, whereas 42% had a change in their employment status. A total of 23% of subjects were requiring support in personal care and in activities of daily living, for 20 h a week or less; only two subjects required more assistance. The description of met and unmet health professional needs is summarized in Fig. 5 and indicates physiotherapy as the most prevalent unmet need.

**Table 4:** Impact on social relations, economic status, and requirement of care in C-ARDS survivors at follow-up

**Social-relational impact**	
Worsening of social relation after your return home	
No	48 (61%)
Yes	30 (38%)
If yes (*N* = 30), have been turned away by the following:	
Family members	5 (17%)
Friends and acquaintances	25 (83%)
**Requirement of care**	
Need for health support	
No	66 (85%)
Yes	12 (15%)
Need for social support	
No	76 (98%)
Yes	2 (2%)
Health/personal care need at home	
No need/autonomy	57 (73%)
< 20 h (weekly)	18 (23%)
20–50 h (weekly)	1 (1%)
> 50 h (weekly)	2 (3%)
**Economic impact**^**a**^	
On active workers (*N* = 33)	
Returned to work	19 (58%)
Gone part-time	8 (24%)
Long-term leave	3 (9%)
Lost work	3 (9%)
On caregivers (*N* = 79)	
No changes	61 (77%)
Gone part-time	4 (5%)
Long-term leave	7 (9%)
Lost work	7 (9%)

^a^Employment changes for patients who were active workers at the time of hospital admission; others (the majority) were retired

## Discussion

The main findings of the present study are as follows: (1) a majority of C-ARDS survivors report primarily physical health-related problems at 6 months after discharge, such as mobility limitation and impairment in the activities of daily life, while anxiety, depression, and other problems related to the mental health were less frequently reported, and not significantly different in comparison with a reference Italian population; (2) two well-validated and internationally used, different sets of HR-QoL questionnaires, the SF-36 and EQ-5D-5L, gave similar results; (3) the risks of developing health-related problems at 6 months after ICU discharge are significantly related with prolonged hospital stay and among people with previous comorbidities, while the level of education and the premorbid income had no impact; and (4) a significant proportion of patients report a worsening of their social relations, experience employment changes, and require health support.

### HR-QoL after COVID-19

In the last decade, an increased awareness towards the long-term consequences of critical illness has been developed, as ensuring good long-term quality of life, rather than simply survive the acute event, has become the main aim of intensive care medicine. Indeed, up to 30% of ICU survivors develop cognitive, physical, and psychological sequelae, independently of the reason for admission [31]. Follow-up of ICU survivors has been suggested to facilitate prompt recognition and treatment of such symptoms and improve long-term physical, psychological, and cognitive outcomes [32]. A total of 80% ARDS survivors experience some degree of cognitive impairment [33–35], as well as a high rate of persistent psychological and physical disability [36] and a reduced HR-QoL [37]. Moreover, viral infections are also characterized by several long-term manifestations [38]. Long-term follow-up of SARS and MERS survivors showed a 30–40% prevalence of post-traumatic stress disorder, depression, anxiety, chronic fatigue, and reduced HR-QoL [39–41]. Like other post-acute viral syndromes, emerging data suggest persistent and prolonged effects after acute COVID-19 [42]. In our cohort, problems in mobility and activities of daily life were reported in about a half of survivors, namely two to three times higher compared to the reference Italian population; compared to norm, impairment in self-care was even more profound (i.e., four times higher). Impressively, only < 25% survivors reported full health. A survey on > 3500 respondents from 56 countries found that systemic and neurological symptoms such as fatigue, cognitive dysfunction, post-exertional malaise, headache, memory troubles, sleep disorders, dizziness, and chest tightness were common and persisting at 6 months after recovery [43]. While still not entirely understood, the underlying mechanism of the long-term neurocognitive consequences of COVID-19 is likely to be multifactorial and might include the direct effects of viral infection, the immunological response, corticosteroid therapy, ICU stay, social isolation, and stigma [44]. Quite interestingly, our results suggest that while previous health state and the severity of the acute illness have a significant impact on the long-term consequences of COVID-19 on health-related quality of life, the level of education and the premorbid income do not.

We found that C-ARDS survivors reported significantly lower HR-QoL domain scores compared to population norms, with the exception of pain (i.e., the most frequently reported problem by normal people [30]) and some of the emotional problems, as assessed by SF-36. Anxiety and depression symptoms were also not significantly different compared to population norms, when using EQ-5D-5L. While it is debated if COVID-19-related acute respiratory failure could be defined as a classic form of ARDS from a pathophysiological point of view [45], in terms of supportive therapy, the disease seems to be similar to the severe forms of classic ARDS, requiring endotracheal intubation, deep sedation, and prolonged mechanical ventilation in the majority of patients. Indeed, the HR-QoL profile observed in our cohort of patients is similar to those observed in non-COVID-19 ARDS survivors [46, 47] or in survivors of ARDS due to other viral infections [48].

As a matter of fact, the physical and psychological sequelae of COVID-19 may even be more frequent than classic ARDS, because of the restriction of visitation and the limitations to social and rehabilitation supports. Of note, only few other studies assessed the HR-QoL in follow-up studies after severe COVID-19 disease. A preliminary report of a 3-month, French follow-up study on 54 ICU survivors found that all patients reported an impaired quality of life, and up to 80% described pain or discomfort in their daily life; almost half complained about mental health disturbances and worsened mobility due to muscular weakness [49]. In a 2-month follow-up in a small cohort of invasively ventilated COVID-19 patients, the overall HR-QoL was reduced to a similar extent as we found, with cognitive and psychological scales showing no impairment, and the most affected dimensions being self-care, usual activities, and pain [50]. In a multicenter cohort mechanically ventilated COVID-19 patients, the HR-QoL at 90 days after ICU discharge was significantly lower than sex- and age-matched random sample population in both physical (mobility, ability to speak, ability to eat) and mental (discomfort, depression, vitality, sexual activity) dimensions [51]. In a 3-month follow-up study, about 75% C-ARDS survivors showed pulmonary functional and structural abnormalities, together with symptoms of dyspnea and cough and a reduced self-reported physical and mental quality of life [10]. Unlike other studies [52], anxiety, depression, and mental problems were far less frequently reported in our cohort of survivors. We wonder whether this might depend on the relatively younger age or prevalent male sex of the patients we enrolled [53], the different tools used for the assessment, or the peculiar characteristic of the national and regional social support system.

### Social and economic burden of recovery after COVID-19 critical illness

To the best of our knowledge, this is the first study to investigate the socio-economic impact of COVID-19-related critical illness. More than 30% of survivors report a worsening of their social relations after returning home, and about 20% require some form of health support during the first 6 months after discharge. About 40% of the patients had not completely returned to their previous employment during the follow-up. This is not unexpected, as a recent systematic review found that about two-thirds of previously employed intensive care unit survivors are jobless up to 3 months following hospital discharge, while those returning to work often experience job loss, occupation change, or worse employment status [54]. As for care received by COVID-19 survivors, this was mainly provided by family members; moreover, about one out of four caregivers reported a change in their employment status. In a UK study, Griffiths and coll. found that one quarter of patients followed up after ICU discharge reported themselves in need of assistance with care at 6 months; reduction in employment and disruption in lifestyle were common in the caregivers, and a negative impact on employment and on reported family earning sources and income was experienced by about 20% of respondents [14].

### Determinants of HR-QoL

The length of hospital stay, and the number of comorbidities, was independently associated with a lower perceived quality of life in ICU survivors. Prolonged mechanical ventilation, ARDS class, and comorbidities are known to be associated with worse HR-QoL in “classic” ARDS survivors [46, 47]. In a recent study, the HR-QoL of severe COVID-19 ICU survivors was significantly associated with age, sex, number of comorbidities, the severity of ARDS, duration of invasive mechanical ventilation, and occupational status, while the marital status and degree of instruction were not [51]. A recent prospective cross-sectional, global, social media online survey assessed > 700 COVID-19 survivors and their caregivers’ health-related quality of life and found how survivors with pre-existing health conditions reported significantly more problems on their physical and psychosocial health [55]. Hypertension and diabetes mellitus are among the most prevalent comorbidities in COVID-19 patients and often coexist as multiple comorbidities, as in our cohort. Age was not associated with worse ICU outcome in our study; an explanation may be the young age of the patients or the small sample size.

Quite unexpectedly, neither education nor the monthly income was found to be associated with any HR-QoL domain. However, these social variables are known to play a significant role in determining the HR-QoL [56], and a recent French investigation found that long-term physical recovery appeared to be poorer in socially deprived ICU survivors [17]. On the one hand, several studies have identified material living conditions (housing and neighborhood), health behaviors, early childhood conditions, and psychosocial stress as important factors underlying the association between social status and health [57]. On the other side, it is also possible that, at 6 months from ICU discharge, the entity of disability related to clinical factors still outweighs the social aspects in determining HR-QoL. Another possible explanation to these apparently conflicting results is that the Italian context (and even more the Autonomous Province of Trento), as compared to that of the other studies, despite worse job indicators and lower average incomes, is characterized by a higher degree of social and family support, a better work-life balance, and an easier access to the health care system, with a lower influence of the education level on life expectancy, which might have counterbalanced the baseline inequality in patient income or education level [58].

### Implications for patient follow-up

These results suggest that patients with severe disease and prolonged hospital stay or more comorbidities may need post-discharge care. While we wait for longer follow-up studies in a larger population, which are necessary to understand the full spectrum and the duration of health consequences from COVID-19, we highlight the need for a multidisciplinary follow-up, involving different healthcare (such as physiotherapists) and social sector professionals (in particular social workers, who played an active role in the management of social fragilities highlighted by the health emergency) [59], able to give integrated answers that are fundamental for an adequate recovery and rehabilitation after COVID-19 critical illness. SF-36 and EQ-5D-5L questionnaires are both valid and complementary tools to identify COVID-19 ICU survivors with persistent symptoms and reduced quality of life.

### Limitations

Several limitations of this study need to be acknowledged; as with any follow-up study, the loss-to-follow-up rate may limit the generalizability of the results; this phenomenon was however comparable to other studies [8, 14, 49, 50]. The patient cohort was relatively small and from only two nearby regional centers, which again may limit the generalizability of the results.

In the current investigation, we included in the HR-QoL assessment of survivors both patients who received invasive mechanical ventilation and patients who only received noninvasive ventilation. Whether this might lead to biased results is still an unresolved issue; in a recent follow-up study on critically ill, C-ARDS patients, 23% of the patients were noninvasively ventilated patients [60]. Latronico et al. assessed 1-year physical, cognitive, and mental health outcomes in a group of critically ill, C-ARDS survivors of whom about 10% only underwent noninvasive ventilation [61]. A similar proportion of noninvasively ventilated patients was included in a HR-QoL investigation in “classic” ARDS survivors [62]. Nevertheless, to account for the presence of both patients who only received noninvasive ventilation, as opposed to patients who were invasively ventilated, we performed two sensitivity analyses, presented in the supplementary materials. We first compared the demographic, socio-economic, and clinical characteristics of the two subgroups of patients, and then we repeated all the analyses only in the subgroup of invasively ventilated patients. Quite interestingly, the two groups had similar demographics and acute physiologic derangements, although noninvasively ventilated patients had lower stays in the ICU and hospital. Albeit our results seem to suggest similar HR-QoL patterns in patients with C-ARDS irrespective of the presence of invasive mechanical ventilation, we caution against such conclusions since the present study is underpowered to address this issue.

Other limitations include the lack of a baseline measurement of HR-QoL before the acute illness and the use of a questionnaire for socio-economic outcomes not previously used in the Italian context. Moreover, we were not able to evaluate potential predictors that may have influenced the reported HR-QoL, such as the dose and the duration of sedatives and neuromuscular blocking agents that have been shown to affect long-term outcomes. COVID-19 pandemic increased unemployment rates and reduced income in general; we lack data for non-COVID-19 controls, and we cannot exclude that the socio-economic effect for both patients and caregivers, employee status and income, is different than for the general population.

## Conclusion

Six months after ICU discharge, a high proportion of patients who had recovered from C-ARDS report a reduced quality of life, due to impaired physical functioning and a delay or inability to return to work; pain, mental health-related problems, and anxiety and depression symptoms were not different in comparison with a reference Italian population. A prolonged hospital and ICU stay and previous comorbidities are associated with health-related problems.

## Supplementary Information

**Additional file 1: Supplementary Table S1.** Demographic, socio-economic and clinical characteristics of invasively and non-invasively ventilated C-ARDS survivors. **Supplementary Table S2.** Univariate analysis of demographics, socio-economic and clinical factors associated with impaired health-related quality of life (EQ-5D-5L) in invasively ventilated C-ARDS survivors. **Supplementary Table S3.** SF-36 domain scores according to demographics, socio-economic and clinical characteristics in invasively ventilated C-ARDS survivors. **Supplementary Figure S1.** Health-related quality of life scale (EQ-5D-5L) in invasively ventilated C-ARDS survivors. **Supplementary Figure S2.** Multivariable analysis of factors associated with impaired “physical” health-related quality of life scale (SF-36 PCS) and “mental” health-related quality of life scale (SF-36 MCS) in mechanically ventilated C-ARDS survivors.

## Data Availability

The datasets used and/or analyzed during the current study are available from the corresponding author on reasonable request.

## Abbreviations

COVID-19: Coronavirus disease 2019
ICU: Intensive care unit
ARDS: Acute respiratory distress disease
C-ARDS: COVID-19-related ARDS
SF-36: 36-Item Short Form Health Survey
EuroQol: European health-related quality of life
HR-QoL: Health-related quality of life
EQ-5D-5L: Five-dimension five-level EuroQol
EQ VAS: EuroQol-Visual Analog Scale
SAPS II: Simplified Acute Physiology Score
PaO_2_: Partial pressure of arterial oxygen
FiO_2_: Inspired fraction of oxygen
LOS: Length of stay
