# Supplementary material for: High rates of impaired quality of life and social and economic problems at 6 months after COVID-19-related ARDS
Source: J Anesth Analg Crit Care. 2022 May 16;2:20. doi: 10.1186/s44158-022-00048-5 (PMC9109430; doi:10.1186/s44158-022-00048-5)
Supplement: Supplementary file 1 — Additional file 1: Supplementary Table S1. Demographic, socio-economic and clinical characteristics of invasively and non-invasively ventilated C-ARDS survivors. Supplementary Table S2. Univariate analysis of demographics, socio-economic and clinical factors associated with impaired health-related quality of life (EQ-5D-5L) in invasively ventilated C-ARDS survivors. Supplementary Table S3. SF-36 domain scores according to demographics, socio-economic and clinical characteristics in invasively ventilated C-ARDS survivors. Supplementary Figure S1. Health-related quality of life scale (EQ-5D-5L) in invasively ventilated C-ARDS survivors. Supplementary Figure S2. Multivariable analysis of factors associated with impaired “physical” health-related quality of life scale (SF-36 PCS) and “mental” health-related quality of life scale (SF-36 MCS) in mechanically ventilated C-ARDS survivors. [file 44158_2022_48_MOESM1_ESM.docx]

**SUPPLEMENTARY MATERIAL**

**High rates of impaired quality of life and social and economic problems at six months after COVID-19 related ARDS**

Michele Umbrello^1^, Sara Miori^2^, Andrea Sanna^2^, Sergio Lassola^2^, Elena Baruzzo^3^, Daniele Penzo^2^, Giovanni Pedrotti^5^, Annamaria Perino^4^, Angelo Colombo^5^, Rocco Pace^2^, Sandra Magnoni^2^

From the:

^1^ Department of Anesthesia and Intensive Care II, San Carlo Borromeo Hospital, ASST Santi Paolo e Carlo; Milano, Italy

^2^ Department of Anesthesia and Intensive Care, Santa Chiara Hospital; Trento, Italy

^3^ Center for Neurocognitive Rehabilitation (CeRiN)- CeRiN-CIMeC, Trento University; Trento, Italy

^4^ Department of Sociology and Social Research, Trento University; Trento, Italy

^5^ Department of Anesthesia and Intensive Care, S. Maria del Carmine Hospital; Rovereto, Italy

Corresponding author and address for reprints:

Dr. Sandra Magnoni

Department of Anesthesia and Intensive Care,

Santa Chiara Hospital,

APSS, Trento, Italy

sandra.magnoni@apss.tn.it

tel 0039 903687

Fax 0039 902692

**Supplementary Table S1.** **Demographic, socio-economic and clinical characteristics of invasively and non-invasively ventilated C-ARDS survivors**

| **Demografic characteristics** | **Invasively**  **ventilated** | **Non- invasively ventilated** |
| --- | --- | --- |
| Number of patients | 69 | 10 |
| Age (years) | 63 (57-71) | 61 (57-63) |
| Sex N |  |  |
| Male | 59 (86%) | 7 (70%) |
| Female | 10 (14%) | 3 (30%) |
| **Socio-economic characteristics** |  |  |
| Family unit composition |  |  |
| Single | 9 (11%) | 2 (10%) |
| ≥ two people | 70 (89%) | 9 (90%) |
| Population of the municipality of residence |  |  |
| < 15.000 | 66 (84%) | 8 (80%) |
| >100.000 | 13 (16%) | 2 (20%) |
| Employment status ^b^ |  |  |
| Active worker | 33 (42%) | 3 (30%) |
| Retired | 43 (54%) | 7 (70%) |
| Monthly Income (€) |  |  |
| < 1500 | 37 (56%) | 4 (44%) |
| > 1500 | 29 (44%) | 5 (56%) |
| Education |  |  |
| ISCED 0-2 | 23 (33%) | 5 (50%) |
| ISCED > 2 | 46 (67%) | 5 (50%) |
| **Clinical characteristics** |  |  |
| No preexisting comorbidities | 35 (51%) | 3 (30%) |
| Preexisting comorbidities | 34 (49%) | 7 (70%) |
| One comorbidity | 19 (28%) | 4 (40%) |
| More than one comorbidity | 15 (21%) | 3 (30%) |
| Comorbidities |  |  |
| Hypertension | 29 (42%) | 7 (70%) |
| Diabetes | 8 (12%) | 1 (10%) |
| Asthma and/or COPD | 9 (13%) | 1 (10%) |
| Ischemic heart disease | 4 (6%) | 2 (20%) |
| Neoplasm | 3 (4%) | 1 (10%) |
| Chronic liver or kidney disease | 0 (0%) | 0 (0%) |
| Immunocompromised | 0 (0%) | 0 (0%) |
| BMI (kg/m^2^) | 27 (25-29) | 30 (28-33) |
| Time from symptoms to hospital admission (days) | 7 (5-10) | 7 (4-15) |
| SAPS II score on ICU admission | 27 (24-33) | 24 (22-30) |
| PaO_2_/FiO_2_ on ICU admission (worst) | 160 (114-225) | 179 (140-205) |
| Renal replacement therapy | 5 (6%) | 0 (0%) |
| Prone positioning | 35 (44%) | 0 (0%) |
| Hydroxychloroquine | 67 (97 %) | 10 (100%) |
| Steroids | 31 (45%) | 6 (60%) |
| Tocilizumab | 11 (16%) | 5 (50%) |
| Tracheostomy | 22 (28%) | 0 (0%) |
| Duration of ventilation (days) | 16 (10-25) | 5 (3-6) |
| ICU LOS (days) | 20 (13-28) | 6 (3-6) |
| Hospital LOS (days) | 40 (29-49) | 25 (16-32) |
| **Laboratory data in ICU** |  |  |
| Creatinine on admission (mg/dl) | 0.92 ± 0.38 | 0.82 ± 0.18 |
| Creatinine max (mg/dl) | 2.00 ± 2.15 | 0.82 ± 0.15 |
| D dimer on admission (µg/mL) | 2407 ± 5892 | 5328 ± 14415 |
| D dimer max (µg/mL) | 5821 ± 9694 | 6031± 14197 |
| WBC on admission (10^3/mL) | 9.47 ± 4.42 | 7.17 ± 2.09 |
| WBC max (10^3/mL) | 17.28 ± 7.6 | 9.20 ± 1.94 |
| CRP on admission (mg/dl) | 113 ± 81 | 130 ± 93 |
| CRP max (mg/dl) | 234 ± 114 | 199 ± 99 |

Values are presented as median (25-75^th^ percentile), mean ± SD, or as count (percentage) for categorical variables. ^b^ Housewife excluded (N=3). Significant differences between mechanically ventilated and non-mechanically ventilated patients are labeld dark-gray; Chi-Square test or Mann-Whitney U test were used for analysis; p< 0.05 was considered significant.

BMI: Body Mass Index~~;~~ SAPS II: Simplified Acute Physiology Score; RRT: renal replacement therapy; ICU: intensive care unit; duration of ventilation: days of ventilation with PEEP > 5 cmH_2_0; LOS: length of stay.

ISCED: International Standard Classification of Education. ISCED 0: Early childhood education (‘less than primary’ for educational attainment), ISCED 1: Primary education, ISCED 2: Lower secondary education, ISCED 3: Upper secondary education, ISCED 4: Post-secondary non-tertiary education, ISCED 5: Short-cycle tertiary education, ISCED 6: Bachelor’s or equivalent level, ISCED 7: Master’s or equivalent level, ISCED 8: Doctoral or equivalent level

WBC: White Blood Cells; CRP: C-Reactive Protein.

**Supplementary Table S2. Univariate analysis of demographics, socio-economic and clinical factors associated with impaired health-related quality of life (EQ-5D-5L) in invasively ventilated C-ARDS survivors.**

| **PAIN OR DISCOMFORT** | | | | |
| --- | --- | --- | --- | --- |
|  | **OR** | **Low 95% CI** | **High 95% CI** | **p value** |
| **Age (yrs)** | 1,03 | 0,62 | 1,72 | 0.904 |
| **Education (ISCED 0-2)** | 1,30 | 0,48 | 3,54 | 0.609 |
| **Income (< 1500)** | 1,17 | 0,44 | 3,09 | 0.758 |
| **Comorbidities (0-1)** | 3,19 | 0,90 | 11,28 | 0.059 |
| **PaO_2_/FiO_2_** | 0,76 | 0,44 | 1,33 | 0.332 |
| **SAPS II** | 0,90 | 0,63 | 1,28 | 0.554 |
| **Ventilation (days)** | 1,01 | 0,63 | 1,62 | 0.964 |
| **Hospital LOS (days)** | 1,30 | 0,93 | 1,80 | 0.108 |
| **MOBILITY** | | | | |
|  | **OR** | **Low 95% CI** | **High 95% CI** | **p value** |
| **Age (yrs)** | 1,39 | 0,82 | 2,35 | 0.216 |
| **Education (ISCED 0-2)** | 1,19 | 0,44 | 3,24 | 0.733 |
| **Income (< 1500)** | 1,02 | 0,38 | 2,68 | 0.976 |
| **Comorbidities (0-1)** | 3,44 | 0,97 | 12,17 | 0.042 |
| **PaO_2_/FiO_2_** | 0,69 | 0,39 | 1,22 | 0.195 |
| **SAPS II** | 1,49 | 1,01 | 2,18 | 0.034 |
| **Ventilation (days)** | 1,74 | 1,03 | 2,95 | 0.029 |
| **Hospital LOS (days)** | 1,80 | 1,25 | 2,60 | 0.002 |
| **USUAL ACTIVITIES** | | | | |
|  | **OR** | **Low 95% CI** | **High 95% CI** | **p value** |
| **Age (yrs)** | 1,85 | 1,05 | 3,25 | 0.017 |
| **Education (ISCED 0-2)** | 1,00 | 0,37 | 2,72 | 1.00 |
| **Income (< 1500)** | 0,41 | 0,16 | 1,06 | 0.070 |
| **Comorbidities (0-1)** | 2,70 | 0,81 | 8,96 | 0.097 |
| **PaO_2_/FiO_2_** | 0,71 | 0,40 | 1,25 | 0.227 |
| **SAPS II** | 1,38 | 0,95 | 2,00 | 0.081 |
| **Ventilation (days)** | 1,51 | 0,92 | 2,49 | 0.095 |
| **Hospital LOS (days)** | 1,59 | 1,10 | 2,30 | 0.006 |
| **ANXIETY AND DEPRESSION** | | | | |
|  | **OR** | **Low 95% CI** | **High 95% CI** | **p value** |
| **Age (yrs)** | 1,03 | 0,60 | 1,78 | 0.904 |
| **Education (ISCED 0-2)** | 1,66 | 0,55 | 5,02 | 0.361 |
| **Income (< 1500)** | 0,70 | 0,24 | 2,03 | 0.512 |
| **Comorbidities (0-1)** | 6,31 | 1,82 | 21,83 | 0.003 |
| **PaO_2_/FiO_2_** | 1,69 | 0,66 | 2,08 | 0.596 |
| **SAPS II** | 1,20 | 0,82 | 1,73 | 0.346 |
| **Ventilation (days)** | 1,12 | 0,68 | 1,83 | 0.661 |
| **Hospital LOS (days)** | 1,27 | 0,92 | 1,75 | 0.147 |
| **SELF CARE** | | | | |
|  | **OR** | **Low 95% CI** | **High 95% CI** | **p value** |
| **Age (yrs)** | 1,72 | 0,88 | 3,35 | 0.096 |
| **Education (ISCED 0-2)** | 1,13 | 0,34 | 3,76 | 0.839 |
| **Income (< 1500)** | 0,65 | 0,19 | 2,20 | 0.482 |
| **Comorbidities (0-1)** | 4,38 | 1,26 | 15,14 | 0.021 |
| **PaO_2_/FiO_2_** | 0,79 | 0,40 | 1,58 | 0.496 |
| **SAPS II** | 1,43 | 0,94 | 2,18 | 0.091 |
| **Ventilation (days)** | 1,38 | 0,80 | 2,37 | 0.248 |
| **Hospital LOS (days)** | 1,57 | 1,08 | 2,27 | 0.013 |

The table shows the results of the univariate analysis of factors associated with problems (of any severity) in each of the five EQ-5D-5L domains (dependent variable). Variables (covariates) significantly (p < 0.05) associated with the development of problems in each single domain are shown as highlighted rows. Reference value for the dependent variable: no problems vs any problems. Covariate values are in rows, with reference values and/or unit of measure, when applicable (factors). Variables were transformed as follows: age/10; PaO2 to FiO2/100; SAPS II/5; ventilation (days)/10; hospital LOS (days)/10.

SAPS II: simplified Acute Physiology Score; LOS: length of stay; PaO_2_/FiO_2_: worst value on the first day of ICU stay; Ventilation: duration of ventilation (days).

**Supplementary Table S3. SF-36 domain scores according to demographics, socio-economic and clinical characteristics in invasively ventilated C-ARDS survivors**

|  |  |  | | ***Physical Function*** | ***Physical Role*** | | ***Bodily***  ***Pain*** | | ***General***  ***Health*** | | ***Vitality*** | | ***Social Function*** | | ***Emotional***  ***Role*** | | ***Mental Health*** | |
| --- | --- | --- | --- | --- | --- | --- | --- | --- | --- | --- | --- | --- | --- | --- | --- | --- | --- | --- |
|  |  |  |  | |  |  | |  | |  | |  | |  | |  | |  |
| **Age (yrs)** | < 65 | 37 | **90** | | **50** | **88** | | **45** | | **65** | | **75** | | **100** | | **80** | |  |
|  |  |  | (80-95) | | (0-100) | (68-100) | | (25-60) | | (55-70) | | (50-88) | | (67-100) | | (72-88) | |  |
|  | >65 | 32 | **83** | | **13** | **79** | | **48** | | **53** | | **63** | | **100** | | **78** | |  |
|  |  |  | (64-95) | | (0-100) | (45-100) | | (20-70) | | (40-71) | | (25-100) | | (67-100) | | (56-88) | |  |
|  | *p* |  | *p=0.2637* | | *p=0.6474* | *p=0.3715* | | *p=0.5792* | | *p=0.3311* | | *p=0.4389* | | *p=0.9940* | | *p=0.5252* | |  |
|  |  |  |  | |  |  | |  | |  | |  | |  | |  | |  |
| **Education**  **(ISCED)** | 0-2 | 23 | **85** | | **0** | **88** | | **50** | | **60** | | **75** | | **100** | | **84** | |  |
|  |  |  | (80-98) | | (0-100) | (68-100) | | (20-70) | | (45-73) | | (50-94) | | (100-100) | | (64-92) | |  |
|  | > 2 | 46 | **85** | | **38** | **78** | | **45** | | **58** | | **63** | | **100** | | **76** | |  |
|  |  |  | (61-95) | | (0-100) | (45-100) | | (25-60) | | (45-70) | | (25-88) | | (33-100) | | (65-88) | |  |
|  | *p* |  | *p=0.3822* | | *p=0.5653* | *p=0.3614* | | *0.5362* | | *p=0.8933* | | *p=0.2874* | | *p=0.1551* | | *p=0.2737* | |  |
|  |  |  |  | |  |  | |  | |  | |  | |  | |  | |  |
| **Monthly**  **Income €** | < 1500 | 37 | **85** | | **0** | **88** | | **45** | | **55** | | **63** | | **100** | | **80** | |  |
|  |  |  | (80-95) | | (0-100) | (55-100) | | (25-60) | | (45-70) | | (38-88) | | (67-100) | | (68-88) | |  |
|  | > 1500 | 29 | **90** | | **75** | **78** | | **55** | | **65** | | **75** | | **100** | | **80** | |  |
|  |  |  | (70-100) | | (0-100) | (55-100) | | (20-75) | | (50-70) | | (50-100) | | (67-100) | | (64-88) | |  |
|  | *p* |  | *p=0.6430* | | *p=0.2115* | *p=0.8530* | | *P=0.1846* | | *p=0.4215* | | *p=0.2770* | | *p=0.8881* | | *p=0.9121* | |  |
|  |  |  |  | |  |  | |  | |  | |  | |  | |  | |  |
| **Comorbidities** | 0-1 | 54 | **88** | | **50** | **90** | | **50** | | **65** | | **81** | | **100** | | **80** | |  |
|  |  |  | (80-99) | | (0-100) | (68-100) | | (30-69) | | (51-74) | | (50-100) | | (100-100) | | (68-88) | |  |
|  | >1 | 15 | **65** | | **0** | **45** | | **20** | | **45** | | **38** | | **0** | | **72** | |  |
|  |  |  | (43-88) | | (0-100) | (23-78) | | (8-45) | | (25-53) | | (13-75) | | (0-100) | | (46-80) | |  |
|  | *p* |  | *p=0.0030* | | *p=0.3682* | *p=0.0004* | | *p=0.0016* | | *p=0.0025* | | *p=0.0037* | | *p=0.0005* | | *p=0.0128* | |  |
|  |  |  |  | |  |  | |  | |  | |  | |  | |  | |  |
| **PaO_2_/FiO_2_ on ICU admission** | <150 | 31 | **80** | | **0** | **78** | | **35** | | **50** | | **63** | | **100** | | **80** | |  |
|  |  |  | (63-95) | | (0-100) | (45-100) | | (20-55) | | (43-70) | | (25-88) | | (17-100) | | (66-88) | |  |
|  | >150 | 38 | **90** | | **75** | **90** | | **50** | | **60** | | **88** | | **100** | | **76** | |  |
|  |  |  | (80-95) | | (0-100) | (55-100) | | (30-74) | | (50-70) | | (50-100) | | (100-100) | | (64-88) | |  |
|  | *p* |  | *p=0.1475* | | *p=0.0998* | *P=0.1278* | | *p=0.0336* | | *p=0.3141* | | *p=0.0435* | | *p=0.1234* | | *p=0.6732* | |  |
|  |  |  |  | |  |  | |  | |  | |  | |  | |  | |  |
| **SAPS II** | < 25 | 22 | **90** | | **63** | **90** | | **43** | | **65** | | **81** | | **100** | | **82** | |  |
|  |  |  | (85-100) | | (0-100) | (68-100) | | (21-59) | | (55-74) | | (53-88) | | (75-100) | | (72-88) | |  |
|  | > 25 | 47 | **85** | | **25** | **78** | | **45** | | **55** | | **63** | | **100** | | **80** | |  |
|  |  |  | (65-95) | | (0-100) | (45-100) | | (23-70) | | (43-70) | | (25-88) | | (50-100) | | (64-88) | |  |
|  | *p* |  | *p=0.0507* | | *p=0.4020* | *p=0.1311* | | *0.8133* | | *p=0.1361* | | *p=0.1088* | | *p=0.3111* | | *p=0.4028* | |  |
|  |  |  |  | |  |  | |  | |  | |  | |  | |  | |  |
| **Ventilation (days)** | <15 | 26 | **90** | | **100** | **90** | | **48** | | **65** | | **84** | | **100** | | **84** | |  |
|  |  |  | (80-100) | | (6-100) | (70-100) | | (25-74) | | (55-78) | | (54-100) | | (100-100) | | (76-88) | |  |
|  | >15 | 43 | **85** | | **0** | **78** | | **45** | | **55** | | **63** | | **100** | | **72** | |  |
|  |  |  | (63-95) | | (0-100) | (48-100) | | (20-60) | | (38-70) | | (31-88) | | (33-100) | | (58-88) | |  |
|  | *p* |  | *p=0.0388* | | *p=0.0043* | *p=0.0672* | | *p=0.0500* | | *p=0.0650* | | *p=0.0187* | | *p=0.0396* | | *p=0.2656* | |  |
|  |  |  |  | |  |  | |  | |  | |  | |  | |  | |  |
| **Hospital LOS (weeks)** | <4 | 16 | **90** | | **100** | **90** | | **45** | | **60** | | **75** | | **100** | | **82** | |  |
|  |  |  | (85-96) | | (0-100) | (89-100) | | (25-68) | | (55-80) | | (50-100) | | (100-100) | | (74-88) | |  |
|  | >4 | 53 | **85** | | **0** | **78** | | **45** | | **63** | | **81** | | **100** | | **80** | |  |
|  |  |  | (60-95) | | (0-100) | (45-100) | | (20-65) | | (40-70) | | (25-88) | | (33-100) | | (60-88) | |  |
|  | *p* |  | *p=0.0295* | | *p=0.0334* | *p=0.0118* | | *p=0.3636* | | *p=0.0497* | | *p=0.0962* | | *p=0.1231* | | *p=0.1708* | |  |

^a^ SF-36 scores for each domain can range from 0 to 100; higher scores correspond to a better health-related quality of life. Data are median (25^th^ -75^th^ percentile) of SF-36 domains score. Data are analized with Mann-Whitney U test; significant differences between groups are labeld dark-gray, p< 0.05 was considered significant.

ISCED: International Standard Classification of Education. ISCED 0: Early childhood education (‘less than primary’ for educational attainment), ISCED 1: Primary education, ISCED 2: Lower secondary education, ISCED 3: Upper secondary education, ISCED 4: Post-secondary non-tertiary education, ISCED 5: Short-cycle tertiary education, ISCED 6: Bachelor’s or equivalent level, ISCED 7: Master’s or equivalent level, ISCED 8: Doctoral or equivalent level; ICU: intensive care unit; SAPS II: Simplified Acute Physiology Score; PaO_2_/FiO_2_: worst value on the first day of ICU stay; Ventilation: duration of ventilation (days); LOS: length of stay.

**Supplementary Figure S1. Health-related quality of life scale (EQ-5D-5L) in invasively ventilated C-ARDS survivors**

**
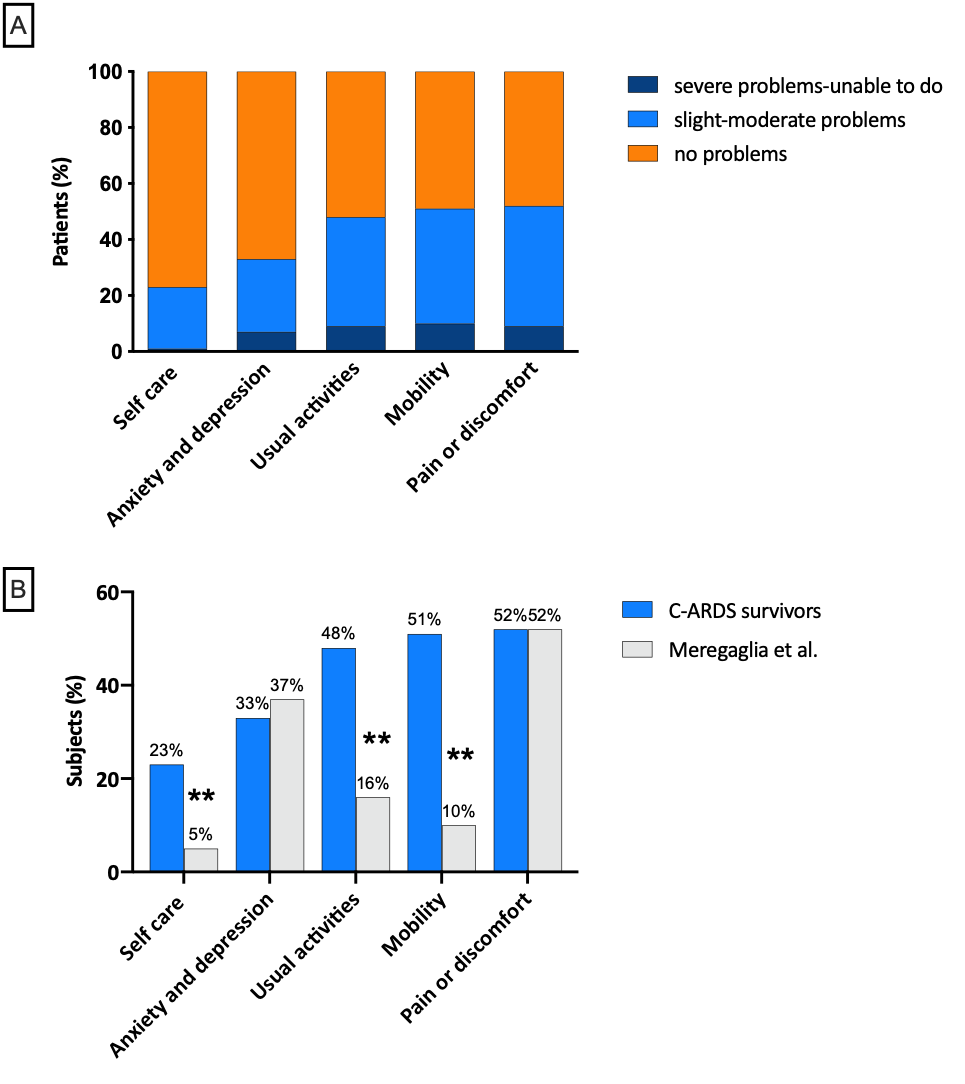
**

A) Frequency distribution of the EQ-5D-5L scores in each of the five domains (pain or discomfort; mobility; usual activities; anxiety and depression; self care). Each domain is scored on a 5-point scale: 1 no problems, 2 slight problems, 3 moderate problems, 4 severe problems, 5 unable to do. Scores are grouped in three classes: no problems; slight to moderate problems; severe problems or unable to do.

B) EQ-5D-5L domain scores for patients responding at the structured interview compared to Italian norm data. Data represent the percentage of subjects (and patients) with problems of any level (2 to 5) in each of the five domains. Significant differences between invasively ventilated C-ARDS survivors (N=69) and age-matched Italian subjects [29] (N=211) were analyzed using Fisher’s exact test. ** p< 0.0001.

**Supplementary Figure S2. Multivariable analysis of factors associated with impaired “physical” health-related quality of life scale (SF-36 PCS) and** **“mental” health-related quality of life scale (SF-36 MCS)** **in mechanically ventilated C-ARDS survivors.**

**
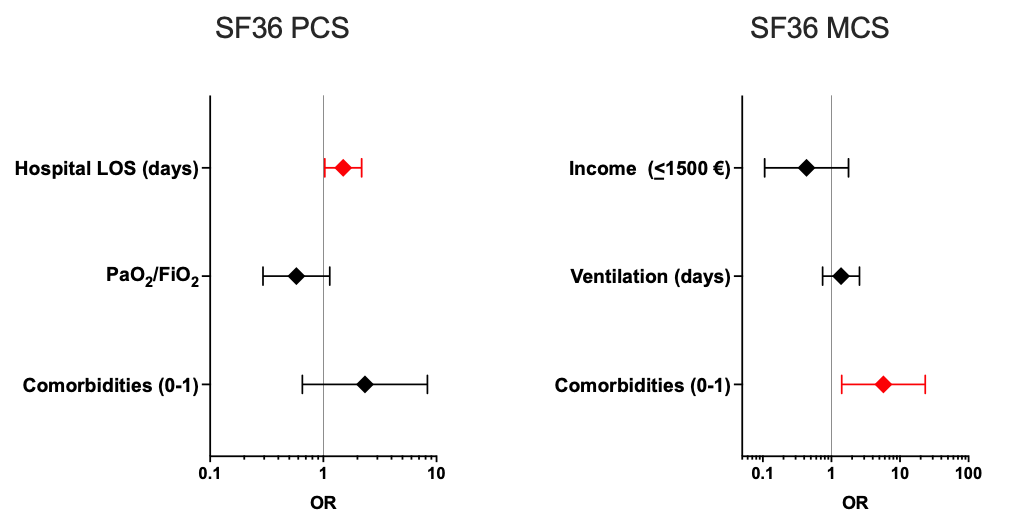
**

The figure shows factors associated with SF-36 physical component summary score (PCS) and SF-36 mental component summary score (MCS) < 40 (dependent variable), the calculated adjusted odds ratios and the 95% confidence intervals, based on the logistic regression, on the X axis and the correspondent forest plot**.** In red, variables significantly associated with an impaired quality of life. Reference values for the dependent variable: PCS and MCS > 40. Covariate values are on the y axis, with reference value, when applicable (factors). Variables were transformed as follows: PaO_2_ to FiO_2_/100; ventilation (days)/10; hospital LOS (days)/10.

LOS: length of stay; PaO_2_/FiO_2_: worst value on the first day of ICU stay; Ventilation: duration of ventilation (days); Comorbidities: no or one comorbidity vs more than one comorbidity.
